# Supplementary material for: Resolving the full spectrum of human genome variation using Linked-Reads
Source: Genome Res. 2019 Apr;29(4):635–45. doi: 10.1101/gr.234443.118 (PMC6442396; doi:10.1101/gr.234443.118)
Supplement: Supplemental Material [file supp_gr.234443.118_Supplemental_longranger-2.2.zip › longranger-2.2.tar.bz2!/lr2.2-public/lib/stan/cmdstan/stan/doc/api/html/agrad_2fwd_2matrix_2mdivide__left__ldlt_8hpp_source.html]

Stan: src/stan/agrad/fwd/matrix/mdivide\_left\_ldlt.hpp Source File


|  |  |
| --- | --- |
|  | Stan  2.5.0  probability, sampling & optimization |


- Main Page
- Related Pages
- Namespaces
- Classes
- Files

- File List
- File Members

All Classes Namespaces Files Functions Variables Typedefs Enumerations Enumerator Friends Macros Pages

- src
- stan
- agrad
- fwd
- matrix

mdivide\_left\_ldlt.hpp

Go to the documentation of this file.

1 #ifndef STAN\_\_AGRAD\_\_FWD\_\_MATRIX\_\_MDIVIDE\_LEFT\_LDLT\_HPP

2 #define STAN\_\_AGRAD\_\_FWD\_\_MATRIX\_\_MDIVIDE\_LEFT\_LDLT\_HPP

3

4 #include <boost/math/tools/promotion.hpp>

5 #include <stan/math/matrix/Eigen.hpp>

6 #include <stan/math/matrix/LDLT\_factor.hpp>

7 #include <stan/math/error\_handling/matrix/check\_multiplicable.hpp>

8 #include <stan/math/matrix/promote\_common.hpp>

9 #include <stan/math/matrix/mdivide\_left\_ldlt.hpp>

10 #include <stan/agrad/fwd/matrix/to\_fvar.hpp>

11

12 namespace stan {

13  namespace agrad {

14

23  template <int R1,int C1,int R2,int C2, typename T2>

24  inline Eigen::Matrix<fvar<T2>,R1,C2>

25  mdivide\_left\_ldlt(const stan::math::LDLT\_factor<double,R1,C1> &A,

26  const Eigen::Matrix<fvar<T2>,R2,C2> &b) {

27  stan::math::check\_multiplicable("mdivide\_left\_ldlt(%1%)",A,"A",

28  b,"b",(double\*)0);

29

30  Eigen::Matrix<T2,R2,C2> b\_val(b.rows(), b.cols());

31  Eigen::Matrix<T2,R2,C2> b\_der(b.rows(), b.cols());

32  for (int i = 0; i < b.rows(); i++)

33  for (int j = 0; j < b.cols(); j++) {

34  b\_val(i,j) = b(i,j).val\_;

35  b\_der(i,j) = b(i,j).d\_;

36  }

37

38  return to\_fvar(mdivide\_left\_ldlt(A, b\_val),

39  mdivide\_left\_ldlt(A, b\_der));

40  }

41  }

42 }

43 #endif

mdivide\_left\_ldlt.hpp

check\_multiplicable.hpp

stan::agrad::fvar

**Definition:** fvar.hpp:13

promote\_common.hpp

stan::agrad::to\_fvar

fvar< T > to\_fvar(const T &x)

**Definition:** to\_fvar.hpp:17

stan::math::check\_multiplicable

bool check\_multiplicable(const char \*function, const T1 &y1, const char \*name1, const T2 &y2, const char \*name2, T\_result \*result)

**Definition:** check\_multiplicable.hpp:15

stan::agrad::mdivide\_left\_ldlt

Eigen::Matrix< fvar< T2 >, R1, C2 > mdivide\_left\_ldlt(const stan::math::LDLT\_factor< double, R1, C1 > &A, const Eigen::Matrix< fvar< T2 >, R2, C2 > &b)

Returns the solution of the system Ax=b given an LDLT\_factor of A.

**Definition:** mdivide\_left\_ldlt.hpp:25

stan::math::LDLT\_factor

**Definition:** LDLT\_factor.hpp:16

Eigen.hpp

to\_fvar.hpp

LDLT\_factor.hpp


---

[ Stan Home Page ]

*© 2011–2014,
Stan Development Team.*
